# Nutritional risk and nutritional status in hospitalized older adults living with HIV in Shenzhen, China: a cross-sectional study

**DOI:** 10.1186/s12879-021-06322-1

**Published:** 2021-06-29

**Authors:** Xiaoning Liu, Jing Cao, Zheng Zhu, Xia Zhao, Jing Zhou, Qiuxia Deng, Liyuan Zhang, Hui Wang

**Affiliations:** 1 The Third People’s Hospital of Shenzhen, Shenzhen, China; 2 Fudan University School of Nursing, Shanghai, China; 3 Fudan University Centre for Evidence-based Nursing: A Joanna Briggs Institute Centre of Excellence, Shanghai, China; 4 Peking Union Medical College Hospital, Beijing, China

**Keywords:** Nutrition status, Nutrition risk, Aging, HIV, AIDS

## Abstract

**Background:**

Nutrition is a crucial factor that can impact morbidity and mortality in older people living with HIV (PLWH). Studies on nutritional risk and nutritional status in all age groups in PLWH have been conducted. However, few studies have focused on nutritional risk in older PLWH. This study aimed to describe the nutritional risk and nutritional status in older PLWH, and explore factors associated with nutritional risk and undernutrition status.

**Methods:**

We conducted a cross-sectional study. We recruited participants aged 50 years or older from the Third People’s Hospital of Shenzhen from January 2016 to May 2019. Nutritional risk and nutritional status were evaluated by the Nutritional Risk Screening 2002 (NRS 2002) tool, body mass index (BMI), albumin level, and prealbumin level on the first day of admission. Logistic regression models were used to identify the factors associated with undernutrition based on the BMI, albumin, and prealbumin criteria.

**Results:**

A total of 196 older PLWH were included in the analysis. We found that 36% of hospitalized older PLWH had nutritional risk, and 12–56% of them had undernutrition based on the BMI, albumin, and prealbumin criteria. An increased nutritional risk score was associated with older age (*β* = 0.265 CI [0.021, 0.096], *P* = 0.002), a higher viral load (*β* = − 0.186 CI [− 0.620, − 0.037], *P* = 0.028), a lower BMI (*β* = − 0.287 CI [− 0.217, − 0.058], *P* = 0.001), and a lower albumin level (*β* = − 0.324 CI [− 8.896, − 1.230], *P* = 0.010). The CD4 count was associated with the prevalence of undernutrition based on the albumin criterion (OR = 15.637 CI [2.742, 89.178], *P* = 0.002).

**Conclusion:**

Our study indicated that nutritional screening, assessment, and management should be routinely performed in hospitalized older PLWH. HIV-specific measures should be used to assess nutritional risk, and albumin, BMI, and other assessments should be used in combination to identify undernutrition in older PLWH.

## Background

HIV is a major public health issue worldwide, and there are nearly 38 million people living with HIV (PLWH) according to the latest report in 2019 from the World Health Organization [1]. Since the development of antiretroviral therapy (ART), PLWH now have a longer life expectancy. The epidemiology of HIV has dramatically changed in the last 20 years, and there is an increasing number of PLWH aged 50 years and older. According to internal reports from the Chinese Center for Disease Control and Prevention, the proportion of PLWH aged 50 years and older was 10.6% in 2003 and increased to 20.2% in 2018 (internal report). Older PLWH face several challenges, including HIV-specific diseases, geriatric syndrome, and age-related functional decline [2, 3].

Among these challenges, nutrition is a crucial factor that can impact morbidity and mortality in older PLWH. Previous studies showed that nutrition can enhance the immune system response to HIV replication and that malnourished PLWH were more likely to develop opportunistic infections than those with adequate nutrition [4, 5]. It is widely believed that malnutrition, the immune system of PLWH, and HIV infection are intertwined and that the former two factors affect the outcome of the latter [5, 6]. Additionally, malnutrition is type of geriatric syndrome. Aging increases the nutritional risk in older adults and eventually leads to increased mortality [7]. The concomitant health conditions, level of activity, and ability to consume food are key factors affecting nutritional risk and nutritional status in older adults [6, 7]. Therefore, maintaining an optimal nutrition status is important for HIV/AIDS management older PLWH.

Screening for nutritional risk and evaluating nutritional status at an early stage allows the implementation of more effective interventions. Nutritional risk is an indicator of prognosis based on nutritional factors [6]. Nutritional risk screening tools, such as the NRS-2002, are very helpful for detecting potential malnutrition [8]. Previous studies reported that the prevalence of nutritional risk in PLWH ranged from 34 to 57% [9–11]. However, the sample sizes of these studies were relatively small and the statistical power was low. Nutritional status in younger PLWH has been widely studied. Measures of nutritional status, including body mass index (BMI), waist circumference, and the levels of albumin, prealbumin, serum glucose, and vitamin D, were commonly used in previous studies [12]. The prevalence of undernutrition in PLWH varies depending on the characteristics of the included populations and the types of nutritional indicators used [13–15]. PLWH in developing countries or rural settings have been reported to have a higher prevalence of undernutrition than PLWH living in developed countries and urban settings [16, 17]. In China, Hu’s study showed that the prevalence of undernutrition ranged from 37.2% when the diagnosis was based on BMI to 77.2% when the diagnosis was based on the malnutrition universal screening tool (MUST) [15].

Studies on nutritional risk and nutritional status have been conducted in PLWH in all age groups. However, few studies have focused on the nutritional risk in older PLWH. The nutritional risk and nutritional status in older PLWH are still unknown. Therefore, the aims of this study were 1) to describe the nutritional risk and nutritional status in older PLWH and 2) to identify factors, including sociodemographic, HIV-related clinical, and nutritional variables, associated with nutritional risk and undernutrition status in older PLWH.

## Methods

### Study design

We conducted a cross-sectional study. Ethics approval for this study was obtained from the Third People’s Hospital of Shenzhen committee on research ethics. Before data collection, written informed consent was prospectively collected from the respondents. Participants at risk of malnutrition were referred to dietary counseling by dieticians to address their specific concerns.

### Study setting

This study recruited participants from the Third People’s Hospital of Shenzhen, which is located in the center of Shenzhen, in southern China. As a first-tier city, Shenzhen has more than 11,800 PLWH, including 5800 patients living with AIDS. The Third People’s Hospital of Shenzhen is a comprehensive hospital providing high-level AIDS care and ART therapy to nearly 12,000 PLWH in the southern area of China since 2004. During the study (January 2016 to May 2019), the Third People’s Hospital of Shenzhen was the only one HIV/AIDS-designated hospital in Shenzhen. Approximately 1205 PLWH received treatment at the HIV/AIDS department for HIV-related comorbidities and ART therapy. Therefore, the subjects enrolled in this study are representative of the older population of PLWH in Shenzhen, China.

### Study participants

Recruitment for this study was conducted over 41 months, from January 2016 to May 2019, by using non-probability sampling. During the study period, there were no major changes in nutritional care practices in the selected departments. All participants who met the following inclusion criteria were eligible for participation: 1) HIV infection was diagnosed by enzyme-linked immunosorbent assay (ELISA) and confirmed by Western blot; 2) hospitalization in the AIDS department in the Third People’s Hospital of Shenzhen; 3) 50 years old and older; and 4) provision of written informed consent. Finally, 196 older PLWH initially participated in this study, 8 of whom (4.1%) were eventually excluded due to missing data.

### Measures

Eligible participants were asked to provide written informed consent. Research nurses collected patient data and administered the survey. Participants received compensation in the form of AIDS care pamphlets. The survey included the following sections.

#### Nutritional risk and nutritional status

Nutritional risk and nutritional status were evaluated by the NRS-2002 tool, body mass index (BMI), albumin level, and prealbumin level on the first day of admission. We used the Chinese version of the NRS-2002 tool to assess nutrition risk in older PLWH [18], which contains three components: 1) nutritional status, body weight changes, and variation in food intake; 2) severity of disease; and 3) age. The NRS-2002 is a widely used tool to predict short-term and long-term risks of malnutrition among hospitalized patients and is recommended by multiple guidelines [19, 20]. The summed score was classified into two categories. A total score of three and above indicated nutritional risk. A total score of five and above indicated a high level of nutritional risk. The instrument had good internal consistency in our sample (Cronbach’s α = .952).

BMI was used to assess participants’ nutritional status, which is a phenotypic criterion for malnutrition diagnosis [19]. BMI was calculated as the weight in kilograms divided by the square of height in meters. The heights and weights of PLWH were measured while they were barefoot and wearing light clothing and were calibrated to within 0.5 cm and 0.5 kg, respectively. For ambulatory participants, height was measured when the subjects were standing erect in the Frankfurt position, and weight was measured with a standing scale. For bedridden participants, height was measured with a tape measure, and weight was measured with a bed scale. BMI was stratified according to the World Health Organization (WHO) criteria: < 17 kg/m^2^ (moderate to severe undernutrition), 17–18.5 kg/m^2^ (mild undernutrition), > 18.5-25 kg/m^2^ (normal nutrition), and > 25 kg/m^2^ (overnutrition).

Albumin and prealbumin, which are traditionally used as markers of the nutritional status of patients, were used to assess nutritional status. Albumin is a strong indicator for diagnosing unexplained weight loss [12]. However it has long half-life and lacks specificity. Prealbumin has shorter half-life than albumin and can reflect acute changes in the nutritional state. Prealbumin decreases when PLWH experience physiological stress, infection, or liver dysfunction [12]. Albumin levels and prealbumin levels were measured via blood tests on the first day of admission. PLWH were defined as having undernutrition if their albumin level was < 35 g/L or prealbumin level was < 200 mg/L.

#### Sociodemographic and clinical variables

Sociodemographic variables such as age, sex, educational attainment, marital status, and employment status were collected with a standard questionnaire. Clinical variables such as duration of HIV positivity, use of ART, ART regimen used, duration of ART, CD4+ T-cell count at admission, viral load at admission, and comorbidities were collected from the medical records.

### Data analysis

Statistical analyses were conducted using SPSS version 22.0 (IBM, Armonk, NY). We used descriptive statistical analyses (e.g., frequencies, percentages, means, and standard deviations) to assess the sociodemographic variables and nutritional status of patients. Linear regression models were used to identify factors associated with the nutritional risk score. All variables were entered into three models at each step. Model 1 included HIV-related variables, including duration of HIV positivity, use of ART (no = 0, yes = 1), ART regimen used, duration of ART, CD4+ T-cell count, viral load, and comorbidities (pneumonia, hepatitis, tuberculosis, diabetes, syphilis, and cancer). Model 2 included nutritional status and sociodemographic variables, including BMI, albumin level (g/L), prealbumin level (mg/L), sex (male = 0, female = 1), age, education attainment (high school or above = 0, otherwise = 1), marital status (married = 0, otherwise = 1), and employment status (employed = 0, otherwise = 1). Model 3 included all the variables. We used three logistic regression models to investigate the factors associated with undernutrition defined according to BMI, albumin, and prealbumin criteria. We considered a two-tailed *P* < 0.05 to indicate statistical significance in all analyses. The Hosmer-Lemeshow test was used to measure the goodness of fit of the logistic regression models. The Akaike and Bayesian Information Criteria (AIC and BIC) were used as estimators of the three logistic models.

## Results

### Participant characteristics, nutritional risk, and nutrition status

This study finally included 196 older PLWH in the analysis. The participants’ characteristics are shown in Table 1. The majority of participants were male (*n* = 174, 88.77%) and currently married (170, 86.73%), with a mean age of 58.41 (SD = 7.99) years. The average duration of HIV positivity was 3.61 (SD = 2.86) years. Fifty-six percent of the participants were receiving ART on admission. Among these participants, most (*n* = 65, 33.16%) were taking TDF-based regimens. The average duration of ART was 3.14 (SD = 2.82) years. Eighty-one percent of the participants had comorbidities.

**Table 1:** Participants characteristics (*N* = 196)

Characteristics	n (%), M ± SD (IQR)		
	Having no nutritional risk n_**1**_ = 125	Having nutritional risk n_**2**_ = 71	Full Sample ***N*** = 196
**Age**	56.58 ± 6.52 (50–83)	61.67 ± 9.26 (50–85)	58.41 ± 7.99 (50–85)
**Gender**			
Male	112 (57.14)	62 (31.63)	174 (88.77)
Female	13 (6.63)	9 (4.59)	22 (11.22)
**Ethnicity**			
Han	125 (63.78)	69 (35.20)	194 (98.98)
Minority	0 (0.00)	2 (1.02)	2 (1.02)
**Education attainment**			
Primary school of below	28 (14.29)	21 (10.71)	49 (25.00)
Secondary school	48 (24.49)	29 (14.80)	77 (39.29)
Post-secondary	40 (20.41)	19 (9.69)	59 (30.10)
University or above	9 (4.59)	2 (1.02)	11 (5.61)
**Marital status**			
Married	113 (57.65)	57 (29.08)	170 (86.73)
Single	12 (6.12)	14 (7.14)	26 (13.27)
**Employment (Yes)**	55 (28.06)	18 (9.18)	73 (37.24)
**Disclosure of HIV Status (Yes)**	78 (39.80)	50 (25.51)	128 (65.31)
**Years since HIV diagnosis**	4.10 ± 3.19 (0–15)	2.69 ± 1.72 (0–15)	3.61 ± 2.86 (0–15)
**Prescribed ART in admission (Yes)**	78 (39.80)	32 (16.33)	110 (56.12)
**Years of ART use**	3.57 ± 3.12 (0–10)	2.31 ± 1.87 (0–10)	3.14 ± 2.82 (0–15)
**ART regimen**			
TDF-based regimen	50 (25.51)	15 (7.65)	65 (33.16)
AZT-based regimen	4 (2.04)	1 (0.51)	5 (2.55)
LPV/r-based regimen	29 (14.80)	11 (5.61)	40 (20.41)
**Presence of comorbidities**	98 (50.00)	61 (31.12)	159 (81.12)
Pneumonia	28 (14.29)	27 (13.78)	55 (28.06)
Hepatitis	7 (3.57)	2 (1.02)	9 (4.59)
Tuberculosis	4 (2.04)	4 (2.04)	8 (4.08)
Diabetes	4 (2.04)	3 (1.53)	7 (3.57)
Syphilis	5 (2.55)	2 (1.02)	7 (3.57)
Cancer	6 (3.06)	0 (0.00)	6 (3.06)
Hypertension	3 (1.53)	0 (0.00)	3 (1.53)
Chronic Diarrheal	0 (0.00)	2 (1.02)	2 (1.02)
**CD4+ T cell count**			
0–50	14 (7.14)	27 (13.78)	41 (20.92)
51–250	62 (31.63)	30 (15.31)	92 (46.94)
251–500	29 (14.80)	12 (6.12)	41 (20.92)
> 501	20 (10.20)	2 (1.02)	22 (11.22)
**Viral load (RNA)**			
< 500	111 (56.63)	68 (34.69)	179 (91.33)
> 500	14 (7.14)	3 (1.53)	17 (8.67)
**Length of in-hospital stay**	11.86 ± 8.90 (1–45)	16.15 ± 10.28 (2–56)	13.42 ± 9.62 (1–56)
**BMI**			
< 17	1 (0.51)	17 (8.67)	18 (9.18)
17–18.5	8 (4.08)	14 (7.14)	22 (11.22)
18.5–25	101 (51.53)	40 (20.41)	141 (71.94)
≥ 25	15 (7.65)	71 (36.22)	15 (7.65)
**Albumin**			
< 35	27 (13.78)	40 (20.41)	67 (34.18)
≥ 35	98 (50.00)	31 (15.82)	129 (65.82)
**Prealbumin**			
< 200	62 (31.63)	58 (29.59)	120 (61.22)
≥ 200	63 (32.14)	13 (6.63)	76 (38.78)

*ART* antiretroviral therapy, *AZT* zidovudine, *BMI* body mass index, *LPV/r* lopinavir–ritonavir, *TDF* tenofovir

Thirty-three percent of the participants (*n* = 71) had a nutritional risk based on the NRS-2002 score. The participants’ BMI values ranged from 14.53 to 29.71, with a mean of 20.90 ± 3.00. Overall, 71.94% of the participants (*n* = 141) had normal nutrition, 9.18% (*n* = 18) had moderate to severe undernutrition, and 11.22% (*n* = 22) had mild undernutrition based on their BMI values. The level of albumin ranged from 13 g/L to 117 g/L, with a mean level of 38.33 ± 9.15 g/L. Based on their albumin levels, 34.18% of the participants (*n* = 67) had undernutrition. The levels of prealbumin ranged from 2 mg/L to 552 mg/L, with a mean level of 182.64 ± 92.69 mg/L. Based on their prealbumin levels, 61.22% of the participants (*n* = 120) had undernutrition.

### Regression analyses

Table 2 shows the results of the multiple linear regression models of the score of nutritional risk in older PLWH. In the final model (*F* = 5.001, *P* = 0.000, *R*^*2*^_*ad*j_ = 0.438), older age (*β* = 0.265 CI [0.021, 0.096], *P* = 0.002), a higher viral load (*β* = − 0.186 CI [− 0.620, − 0.037], *P* = 0.028), a lower BMI (*β* = − 0.287 CI [− 0.217, − 0.058], *P* = 0.001), and a lower albumin level (*β* = − 0.324 CI [− 8.896, − 1.230], *P* = 0.010) were associated with an increased nutritional risk score.

**Table 2:** Multiple logistic regression models of nutrition risk in older PLWH (N = 196)

Variables	Model 1		Model 2		Model 3	
	***β (CI)***	***P***	***β (CI)***	***P***	***β (CI)***	***P***
**BMI**			−0.395 (− 0.248, − 0.123)	0.000	− 0.287 (− 0.217, − 0.058)	0.001
**Lg (Albumin)**			− 0.311 (−7.598, −2.465)	0.000	− 0.324 (−8.896, − 1.230)	0.010
**Lg (Prealbumin)**			− 0.003 (− 0.813, 0.781)	0.969	0.057 (− 1.104, 1.789)	0.639
**Male**			0.024 (− 0.478, 0.694)	0.717	0.059 (− 0.400, 0.891)	0.452
**Age**			0.245 (0.023, 0.083)	0.001	0.265 (0.021, 0.096)	0.002
**Married**			0.073 (−0.246, 0.930)	0.252	0.091 (−0.273, 1.803)	0.238
**Secondary school or below**			−0.030 (− 0.491, 0.306)	0.647	− 0.096 (− 0.808, 0.210)	0.246
**Employed**			0.098 (−0.111, 0.708)	0.152	0.082 (−0.238, 0.723)	0.319
**Year since HIV diagnosis**	−0.190 (− 0.326, 0.127)	0.384			−0.038 (− 0.232, 0.192)	0.851
**ART regimen**						
TDF-based regimen	0.057 (−0.457, 0.812)	0.580			0.038 (−0.404, 0.643)	0.652
AZT-based regimen	0.121 (−0.492, 2.347)	0.198			0.148 (−0.059, 2.314)	0.062
LPV/r-based regimen	0.013 (−0.654, 0.742)	0.900			−0.022 (− 0.646, 0.490)	0.786
**Years of ART use**	0.012 (−0.226, 0.239)	0.955			−0.091 (− 0.261, 0.164)	0.652
**Comorbidities**						
Pneumonia	−0.160 (−1.216, 0.153)	0.126			−0.126 (−1.001, 0.163)	0.156
Hepatitis	−0.031 (− 1.260, 0.890)	0.734			−0.054 (−1.229, 0.599)	0.495
Tuberculosis	−0.154 (−2.189, 0.245)	0.116			−0.120 (−1.752, 0.242)	0.136
Diabetes	−0.015 (−1.385, 1.186)	0.878			−0.088 (−1.669, 0.465)	0.265
Syphilis	0.115 (−0.594, 2.172)	0.260			0.098 (−0.485, 1.836)	0.251
Cancer	0.098 (−0.773, 2.494)	0.298			0.084 (−0.624, 2.094)	0.286
**Lg (CD4 count)**	−0.209 (−1.066, 0.025)	0.061			−0.041 (− 0.586, 0.384)	0.680
**Lg (RNA)**	−0.181 (− 0.672, 0.031)	0.074			−0.186 (− 0.620, − 0.037)	0.028

Model 1: *F* = 2.290, *P* = 0.011, *R*^*2*^_*ad*j_ = 0.134. Hypertension, and chronic diarrheal were excluded due to few participants

Model 2: *F* = 14.481, *P* = 0.000, *R*^*2*^_*ad*j_ = 0.433

Model 3: *F* = 5.001, *P* = 0.000, *R*^*2*^_*ad*j_ = 0.438

Table 3 shows the results of the multiple logistic regression models of nutritional status in older PLWH. In Model 1 (*P* = 0.997, AIC = 67.941, BIC = 107.278), no factors were associated with undernutrition based on BMI in older PLWH (*P* > 0.05). In Model 2 (*P* = 0.496, AIC = 151.766, BIC = 197.670), a lower CD4+ count was associated with undernutrition based on albumin (OR = 15.637 CI [2.742, 89.178], *P* = 0.002). In Model 3 (*P* = 0.943, AIC = 152.969, BIC = 202.141), older age was associated with undernutrition based on prealbumin (OR = 0.892 CI [0.800, 0.993], *P* = 0.038).

**Table 3:** Logistic regression model of nutrition status in older PLWH (*N* = 196)

Variables	Undernutrition based on BMI criteria		Undernutrition based on albumin criteria		Undernutrition based on prealbumin criteria	
	OR (CI)	***P***	OR (CI)	***P***	OR (CI)	***P***
**Male**	1.627 (0.189–13.990)	0.657	2.527 (0.355–17.989)	0.355	2.058 (0.354–11.956)	0.421
**Age**	0.926 (0.828–1.037)	0.183	0.963 (0.863–1.074)	0.497	0.892 (0.800–0.993)	0.038
**Married**	2.097 (0.239–18.427)	0.504	0.088 (0.005–1.630)	0.103	3.963 (0.444–35.377)	0.218
**Secondary school or below**	0.148 (0.012–1.750)	0.129	1.675 (0.329–8.535)	0.535	1.906 (0.580–6.261)	0.288
**Employed**	0.929 (0.121–7.138)	0.943	0.719 (0.132–3.916)	0.703	1.297 (0.459–3.667)	0.624
**Year since HIV diagnosis**	0.992 (0.524–1.876)	0.979	1.132 (0.607–2.114)	0.696	1.129 (0.765–1.666)	0.542
**ART regimen**						
TDF-based regimen	–	–	–	–	0.521 (0.168–1.618)	0.259
AZT-based regimen	–	–	–	–	0.416 (0.030–5.696)	0.511
LPV/r-based regimen	–	–	–	–	1.401 (0.358–5.483)	0.628
**Years of ART use**	1.218 (0.558–2.656)	0.077	1.266 (0.601–2.666)	0.534	0.964 (0.626–1.483)	0.867
**Comorbidities**						
Pneumonia	0.512 (0.087–3.009)	0.459	0.986 (0.161–6.057)	0.988	0.298 (0.054–1.662)	0.168
Hepatitis	0.130 (0.006–2.629)	0.183	0.041 (0.001–1.392)	0.076	0.691 (0.087–5.151)	0.727
Tuberculosis	0.031 (0.001–1.274)	0.067	1.803 (0.005–47.014)	0.846	–	–
Diabetes	–	–	4.048 (0.241–27.957)	0.331	–	–
Syphilis	–	–	0.091 (0.002–4.279)	0.223	1.319 (0.095–18.312)	0.837
**Lg (CD4 count)**	0.912 (0.170–4.879)	0.914	15.637 (2.742–89.178)	0.002	3.540 (0.851–14.718)	0.082
**Lg (RNA)**	0.754 (0.306–1.862)	0.541	2.693 (0.722–10.046)	0.140	1.302 (0.638–2.656)	0.469

Model 1 and Model 2: TDF-based regimen, AZT-based regimen, and LPV/r-based regimen were excluded due to few participants in both groups;

Model 3: Tuberculosis and diabetes were excluded due to few participants in both groups;

Model 1: *P* = 0.997, AIC = 67.941, BIC = 107.278; Model 2: *P* = 0.496, AIC = 151.766, BIC = 197.670; Model 3: *P* = 0.943, AIC = 152.969, BIC = 202.141

## Discussion

Our study provides an assessment of nutritional risk and status in hospitalized older PLWH, as evaluated by the NRS-2002 tool, BMI, albumin level, and prealbumin level. To the best of our knowledge, this is the first study to explore the prevalence of nutritional risk and undernutrition in hospitalized older PLWH and identify factors associated with nutritional risk and status as defined by the BMI, albumin and prealbumin criteria. The findings of the present study can help healthcare professionals identify hospitalized older PLWH with nutritional risk and provides empirical evidence that can be used when developing strategies to address undernutrition and wasting.

The results indicated that the prevalence of nutritional risk in hospitalized older PLWH in our study was nearly 20% lower than that reported in other studies conducted in PLWH in all age groups [15–17]. The explanation may lie in the differences in the characteristics of the sample populations, including the severity of comorbidities, viral load, and type of ART. Unfortunately, healthcare professionals are unaware of the fact that 30–50% of older PLWH are in danger of malnutrition [21, 22]. Additionally, medical nutritional screening and interventions are not included in most HIV/AIDS treatment and management guidelines in China. Malnutrition and energy deficiency management, including nutritional screening, assessment, and management, should be recommended as routine practice for hospitalized older PLWH.

Additionally, in our study, we found that the severity of nutritional risk was associated with the viral load. This study used the NRS-2002 to evaluate nutritional risk in hospitalized older PLWH. Although the NRS-2002 is a widely used tool in PLWH, some HIV-specific variables, such as viral load, are not included in this measurement tool [9, 23, 24]. In particular, HIV or AIDS, which is regarded as a chronic wasting disease, is also not included in first domain of the NRS-2002 [25]. Therefore, using the NRS-2002 may underestimate the prevalence of nutritional risk. The results indicated that validated disease-specific nutritional screening tools are needed.

The findings from our study showed that undernutrition is common in hospitalized older PLWH in China, and 12–56% of older PLWH have undernutrition as defined by different criteria. The prevalence of undernutrition was lower than those reported in previous studies conducted in all age groups of PLWH [9–11, 26]. Hu and colleagues surveyed 94 PLWH in Chengdu, China, and found that 21.3–77.2% had undernutrition as defined by the BMI, Subjective Global Assessment (SGA), and Malnutrition Universal Screening Tool (MUST) criteria [15]. Birhane’s and Hailemariam’s studies found that the prevalence of undernutrition in PLWH in Ethiopia was 25.2 and 12.3%, respectively, as defined by the BMI criteria [16, 17]. Other studies conducted in Brazil, Indonesia, and Morocco showed that 12.3 to 36.1% of PLWH met the BMI criteria for undernutrition [9, 13, 27]. However, few studies have been conducted to explore nutritional status in older PLWH. A previous study found that aging and aging-related comorbidities could affect nutritional status in older adults [28]. The reasons for the relatively better nutritional status reported in our study may be as follows: 1) our sample population had higher CD4 counts than those in previous studies. Higher CD4 counts are associated with a lower probability of developing an opportunistic infection [29, 30]. Additionally, in our study, the CD4 count was significantly associated with undernutrition as defined by the albumin criteria and nearly significantly associated with undernutrition as defined by the prealbumin criteria. Therefore, PLWH with higher CD4 counts may have a better nutritional status. 2) Our sample population was from an urban setting in a first-tier city in China. Urban residents are expected to have a greater food intake than those who live in rural regions [16, 17].

Additionally, undernutrition as defined by different criteria had different associated factors. A BMI < 18.5 kg/m^2^ is a widely used indicator of undernutrition in PLWH and the elderly population. A low BMI after receiving ART is also associated with increased mortality in PLWH [31]. In our study, we did not identify any factors that were associated with undernutrition as defined by the BMI criteria, which indicated that these criteria may not truly reflect the processes occurring in older PLWH. This opinion is consistent with that expressed in Ruiz’s review, which suggested that the waist-to-hip ratio was a better option than BMI for evaluating undernutrition status [32]. Albumin and prealbumin are two major laboratory indicators used to evaluate undernutrition. We found that undernutrition as defined by the prealbumin criteria was too sensitive in older PLWH due to its short half-life. Among the three sets of criteria used to define undernutrition, the albumin-based criteria were the best marker in older PLWH. The CD4 count was associated with the prevalence of undernutrition as defined by the albumin criteria, which was supported by the findings of previous studies [33, 34]. However, based on our clinical experience, a reduced albumin level may not necessarily indicate poor nutrition but may be due to acute pathophysiologic events caused by the depletion of CD4 cells [35]. Therefore, measuring albumin alone cannot clearly reflect the nutritional status in older PLWH. Albumin, BMI, and other assessments should be used in combination to identify undernutrition among older PLWH.

## Limitations

This is the first study exploring the prevalence of nutritional risk and undernutrition in hospitalized older PLWH, and some limitations exist. First, the sample size of our study was relatively small, and the patients were recruited from only one hospital. Large-scale and multicenter studies are needed to increase the statistical power of the analyses and provide external validity. Second, we included only older hospitalized PLWH who voluntarily participated in this study. The prevalence of undernutrition may be underestimated due to the presence of older hospitalized PLWH with severe conditions who were unable to participate in this study. Third, the nutritional risk may have been underestimated due to the use of non-disease-specific measurement tools. Fourth, the study was cross-sectional in nature, and the results do not establish the directionality of the associations. Future studies need to use HIV-specific measures such as the Rapid Nutritional Screening for HIV disease tool (RNS-H) or multiple nutritional risk screening measures.

## Conclusion

Our study generated evidence regarding the prevalence of nutritional risk and undernutrition in hospitalized older PLWH in China. We found that 36% of hospitalized older PLWH had a nutritional risk, and 20–61% of older PLWH had undernutrition as defined by the BMI, albumin, and prealbumin criteria. Our study indicated that nutritional screening, assessment, and management should be routinely performed for hospitalized older PLWH. HIV-specific measures are recommended for the assessment of nutritional risk, and criteria based on albumin, BMI, and other measures should be used together to identify undernutrition among older PLWH.

## Data Availability

The datasets used or analyzed during this study are available from the corresponding author on reasonable request.

## Abbreviations

HIV: Human Immunodeficiency Virus
AIDS: Acquired Immune Deficiency Syndrome
PLWH: Patients living with HIV
NRS: Nutritional risk screening
BMI: Body Mass Index
MUST: Malnutrition Universal Screening Tool
ART: Antiretroviral therapy
AZT: Zidovudine
LPV/r: Lopinavir–ritonavir
TDF: Tenofovir
SGA: Subjective Global Assessment

## References

[1] World Health Organization. Progress report on HIV, viral hepatitis and sexually transmitted infections 2019: accountability for the global health sector strategies, 2016–2021. Geneva: World Health Organization; 2019.

[2] He N, Ding Y, Li J, Yuan S, Xu L, Qiao S, Xu X, Zhu B, Shi R, Barile JP, Wong FY (2019). HIV and aging in mainland China: implications for control and prevention research. Curr HIV/AIDS Rep.

[3] Zhu Z, Hu Y, Xing W, Guo M, Wu B (2019). Perceived discrimination and cognitive function in middle-aged and older adults living with HIV in China. AIDS Care.

[4] Kourkouta L, Monios A, Iliadis C, Ouzounakis P (2017). AIDS and nutrition in patients. Progress Health Sci.

[5] Duggal S, Chugh TD, Duggal AK (2012). HIV and malnutrition: effects on immune system. Clin Dev Immunol.

[6] Mussi C (2016). Nutrition and physical exercise in older patients with HIV. Managing the older adult patient with HIV.

[7] Díaz Ramos JA, González Hernández LA, Fraga Ávila C, Asencio-del Real G, Piñeirúa-Menéndez A, Leal-Mora D, Ávila-Funes JA (2016). Nutritional issues in geriatric care: nutrition and HIV. J Lat Am Geriatric Med.

[8] Kondrup J, Rasmussen HH, Hamberg OL, STANGA Z (2003). An ad hoc ESPEN working group. Nutritional risk screening (NRS 2002): a new method based on an analysis of controlled clinical trials. Clin Nutr.

[9] Costa CS, Neto CL, Câmpelo WF, Mendes AL (2017). Association between different methods of nutrition assessment in patients with HIV/AIDS in a public hospital. Rev Bras PromoçãoSaúde.

[10] Wright L, Konlan MB, Boateng L, Epps JB (2019). Nutrition risk and validation of an HIV disease-specific nutrition screening tool in Ghana. South Afr J Clin Nutr.

[11] Yongzhan Z, Ping M, Jianfeng Z, Jing W (2013). Application of NRS 2002 for nutritional screening in patients with AIDS. Chongqing Med.

[12] Keller U (2019). Nutritional laboratory markers in malnutrition. J Clin Med.

[13] El Alama H, Fatihi T, Benmoussa A, Barkat A (2020). Assessment of nutritional status in HIV-positive population. Adv Tradition Med.

[14] Lazzari TK, Forte GC, Silva DR (2018). Nutrition status among HIV-positive and HIV-negative inpatients with pulmonary tuberculosis. Nutr Clin Pract.

[15] Hu W, Jiang H, Chen W, He SH, Deng B, Wang WY, Wang Y, Lu CD, Klassen K, Zeng J (2011). Malnutrition in hospitalized people living with HIV/AIDS: evidence from a cross-sectional study from Chengdu, China. Asia Pac J Clin Nutr.

[16] Hailemariam S, Bune GT, Ayele HT (2013). Malnutrition: prevalence and its associated factors in people living with HIV/AIDS, in Dilla University referral hospital. Arch Public Health.

[17] Birhane M, Loha E, Alemayehu FR. Nutritional status and associated factors among adult HIV/AIDS patients receiving ART in Dilla University referral hospital, Dilla, Southern Ethiopia. J Med Physiol Biophys. 2021;70:8–15.

[18] Kondrup JE, Allison SP, Elia M, Vellas B, Plauth M (2003). ESPEN guidelines for nutrition screening 2002. Clin Nutr.

[19] Cederholm T, Jensen GL, Correia MI, Gonzalez MC, Fukushima R, Higashiguchi T, Baptista G, Barazzoni R, Blaauw R, Coats AJ, Crivelli AN (2019). GLIM criteria for the diagnosis of malnutrition–a consensus report from the global clinical nutrition community. J Cachexia Sarcopenia Muscle.

[20] McClave SA, Taylor BE, Martindale RG, Warren MM, Johnson DR, Braunschweig C, McCarthy MS, Davanos E, Rice TW, Cresci GA, Gervasio JM (2016). Guidelines for the provision and assessment of nutrition support therapy in the adult critically ill patient: Society of Critical Care Medicine (SCCM) and American Society for Parenteral and Enteral Nutrition (ASPEN). JPEN. J Parenter Enter Nutr.

[21] Tafadzwa D, Gashema P, Michael H, Rosemary O. Nutrition and HIV/AIDS: A qualitative study on perceived factors affecting feeding practices among adult people living with HIV/AIDS in Kigali, Rwanda. Nutri Food Sci Int J. 2019;9(4):555767.

[22] Sackey J, Zhang FF, Rogers B, Aryeetey R, Wanke C. Implementation of a nutrition assessment, counseling and support program and its association with body mass index among people living with HIV in Accra, Ghana. AIDS care. 2018;30(5):586–90. 10.1080/09540121.2017.1420137. 10.1080/09540121.2017.1420137 PMC6067919 29284281

[23] Blaauw R, Achar E, Dolman RC, Harbron J, Moens M, Munyi F, Nyatefe D, Visser J (2019). The problem of hospital malnutrition in the African continent. Nutrients.

[24] Sangarlangkarn A, Apornpong T, Justice AC, Avihingsanon A (2019). Screening tools for targeted comprehensive geriatric assessment in HIV-infected patients 50 years and older. Int J STD AIDS.

[25] Baum MK, Tamargo JA, Wanke C (2020). Nutrition in HIV and tuberculosis. Nutrition and infectious diseases.

[26] Teshome MS, Gissa SB, Tefera BZ, Lema TB (2017). Undernutrition and its predictors among people living with HIV/AIDS attending antiretroviral therapy clinic in Jimma University specialized hospital. Int J Nutr Metabol.

[27] Putri CL, Siregar ML, Harapan H, Husnah H (2018). Malnutrition and opportunistic infections among people living with HIV receiving anti-retroviral therapy in Aceh, Indonesia. Iran J Public Health.

[28] Zhu Z, Hu Y, Xing W, Guo M, Zhao R, Han S, Wu B (2019). Identifying symptom clusters among people living with HIV on antiretroviral therapy in China: a network analysis. J Pain Symptom Manag.

[29] Fonsah JY, Njamnshi AK, Kouanfack C, Qiu F, Njamnshi DM, Tagny CT, Nchindap E, Kenmogne L, Mbanya D, Heaton R, Kanmogne GD (2017). Adherence to antiretroviral therapy (ART) in Yaoundé-Cameroon: association with opportunistic infections, depression, ART regimen and side effects. PLoS One.

[30] Chatterjee S, Akbar F, Das N, Ray K, Bandyopadhyay A, Singh MK (2016). Quality of life of HIV/AIDS patients: the influence of CD4 count on it. Natl J Commun Med.

[31] Jiang J, Qin X, Liu H, Meng S, Abdullah AS, Huang J, Qin C, Liu Y, Huang Y, Qin F, Huang J (2019). An optimal BMI range associated with a lower risk of mortality among HIV-infected adults initiating antiretroviral therapy in Guangxi, China. Sci Rep.

[32] Ruiz M, Kamerman LA (2010). Nutritional screening tools for HIV-infected patients: implications for elderly patients. J Int Assoc Phys AIDS Care.

[33] Pralhadrao HS, Kant CH, Phepale KI, Mali MK (2016). Role of serum albumin level compared to CD4+ cell count as a marker of immunosuppression in HIV infection. Ind J Basic Appl Med Res.

[34] Obiako RO, Akase IE, Hassan A, Babadoko A, Musa BO, Balogun Y, Okonkwo L, Yusuf R, Muktar HM, Obadaki M, Abdu-Aguye I (2019). Malnutrition in HIV: are patients in early stages of disease and with high CD4 counts spared?. Sub-Saharan Afr J Med.

[35] Smith SH (2017). Using albumin and prealbumin to assess nutritional status. Nursing.

